# Quality Characteristics of Black Soldier Flies Produced by Different Substrates

**DOI:** 10.3390/insects14060500

**Published:** 2023-05-29

**Authors:** Abdolreza Hosseindoust, Sang Hun Ha, Jun Young Mun, Jin Soo Kim

**Affiliations:** Department of Animal Industry Convergence, Kangwon National University, Chuncheon 24341, Republic of Korea

**Keywords:** conversion ratio, digestibility, fatty acids, food waste, sustainability, tofu, vegetable

## Abstract

**Simple Summary:**

Sustainable agriculture endeavors to achieve two primary objectives, namely, the provision of nourishing food and the mitigation of detrimental environmental impacts. The black soldier fly, known as *Hermetia illucens*, has emerged as a promising solution for sustainable agriculture in recent years. Nonetheless, it is essential to investigate the nutritional prerequisites of black soldier flies, contingent on the source of their diets, such as tofu by-products, food waste, and vegetables, to optimize the conversion ratio. Accordingly, this study aimed to evaluate the efficacy of tofu by-products, food waste, and vegetables in terms of promoting black soldier fly growth and conversion efficiency. The findings indicate that tofu by-products can be suitably employed for fostering larval growth and nutrient accumulation.

**Abstract:**

Black soldier fly (BSF; *Hermetia illucens*) has a high capacity for amino acids and fatty acid accumulation. This study was conducted to assess the effectiveness of tofu by-products, food waste, and vegetables for BSF growth and conversion efficiency. BSFs under tofu by-product treatments showed the greatest weight at d 12 and the harvest period. Moreover, BSF larval weight was greater in the food waste treatment compared with the vegetable treatment at d 12 and harvest. The larva yield result was greater in the vegetable treatment compared with the tofu by-product. The bioconversion rate was higher in the tofu by-product treatment compared with the food waste and vegetable treatments. The protein conversion rate and lipid conversion rate were the highest in the vegetable treatment. The protein yield and lipid yield were greatest in the tofu by-product treatment. The lauric acid content was increased in BSFs fed tofu by-products compared with the food waste treatment. The concentration of C16:1 was the highest in the tofu by-product treatment. The content of oleic acid and α-linolenic acid was higher in BSFs fed tofu by-products compared with the vegetable treatment. In conclusion, the tofu by-products show benefits for larval growth and nutrient accumulation, which can improve larval quality for livestock feed ingredients.

## 1. Introduction

Sustainable agriculture aims to provide nutritious food while minimizing negative impacts on the environment [1,2,3]. In addition, the cost of feed ingredients has been steadily increasing as a result of population growth. Furthermore, the global fishing industry is facing a growing concern over the over-harvesting of fish for fishmeal production, as evidenced by references [4,5,6]. As such, there is a need for protein sources, including insects, algae, and mycoprotein, that are environmentally friendly and efficient [7,8]. The black soldier fly (BSF; *Hermetia illucens*) has gained attention in recent years as a potential solution for sustainable agriculture [9,10]. The larvae of this species have been shown to have several benefits as a protein source, making them an attractive option for animal nutrition, thus addressing food security challenges [1,11]. One of the main benefits of BSF production is its high efficiency in converting organic waste into protein [7,12,13]. The larvae of this species are capable of breaking down a wide variety of organic materials, including animal manure, food waste, and agricultural residue, into high-protein biomass [7,13,14]. This ability to convert waste into protein makes BSF production an attractive solution for reducing waste and increasing the sustainability of animal feed production. Another benefit of BSF production is the low environmental impact [15]. Unlike traditional livestock production, BSF production has a lower carbon footprint and requires less water [15,16]. Additionally, the larvae of this species produce fewer greenhouse gases compared with other livestock, making BSF production a more environmentally friendly option [17].

In terms of economic benefits, BSF production is cost-effective compared with traditional livestock production [4,18]. The larvae can be raised on organic waste and other low-cost feedstocks, reducing the cost of production [13,17,19]. The larvae of the BSF are capable of breaking down a wide variety of organic materials, including food waste and vegetables, into high-protein biomass [7,18]. Tofu by-products are also another feed alternative for BSF production, particularly in that the main by-product of processed tofu is often treated as waste. Tofu production generates large amounts of waste, including tofu whey, which is typically considered low value and is often discarded [17,20]. Tofu by-products are used in just a few nations, such as Republic of Korea, Japan, and China, with approximately 14 million tons of annual waste [17,20]. This ability to convert waste into protein makes BSF production an attractive solution for reducing waste and increasing the sustainability of animal feed production. Additionally, the larvae can be raised in a closed system, reducing the risk of environmental contamination and promoting more efficient use of resources [4,20,21]. The use of different botanical waste products as feed for BSF larvae can significantly affect their growth performance, conversion efficiency, and larval quality. Optimizing their diet can lead to increased protein production per kg of food waste, making BSF production a sustainable and cost-effective solution for animal feed production while reducing waste and environmental impacts. Thus, it is important to evaluate the performance of BSFs with different food waste in order to maximize protein production per kg of food waste. This study was conducted to evaluate the BSF’s growing performance, convergence efficiency, and quality of larvae using different botanical waste to search for its optimal diet.

## 2. Materials and Methods

### 2.1. Larvae Rearing Control

This trial was conducted at a commercial BSF farm located in Eui-sung, Gyeongsangbuk-do, Republic of Korea. The insects used in this study (BSF; *Hermetia illucens*) were supplied before hatching. The trial colony was maintained under controlled conditions (temperature: 27 ± 0.2 °C; relative humidity: 65 ± 10%; 16 h photophase natural light supplemented with LED light at 6000 lux). A total of 30 clutches (containing 50,000 larvae, 24.12 ± 0.65 mg per egg block) were used in this study. Average egg weight was 0.48 µg. There were 3 treatments, tofu by-products, food waste, and vegetables (cabbage, radish, lettuce, and carrot), with 10 replicates per treatment with a dimension of 1064 × 507 × 320 cm (length × width × height) for each experimental box. The trial was continued to the end of the prepupa stage and before the pupa stage. The method for counting eggs in a clutch was based on Georgescu et al. [21], using an Alpha model binocular magnifier with 7× to 45× magnification. The eggs were dispersed with 70% ethanol and then captured and counted through photographic means with the aid of the ClickMaster2000 1.0 software, which is available at https://www.thregr.org/~wavexx/ (accessed on 20 January 2023).

### 2.2. Larval Growing Performance

To evaluate larval growth performance, larvae were collected at d 14, and weight, length, width, and survivability were measured. To compare development days, 100 larvae were randomly collected starting on d 10 until the average weight reached 190 mg (average weight of food waste treatment). The larval yield reached larvae survivability on d 14 of the trial, based on the following equation [21].

Larval yield % = Larvae_F_/Larvae_I_ × 100

where Larvae_F_ represents the number of larvae at the end of the trial, and Larvae_I_ represents the number of larvae at the initiation of the trial.

### 2.3. Substrates

The substrates used in this trial were supplied by local farms and facilities. Water was sprayed on dried substrates to increase the moisture to 65%. Before hatching, egg blocks were located on 3 different substrates (food waste, tofu by-products, and vegetables). Food waste was gathered at a local environmental waste-collecting facility (Eui-sung, Gyeong-sangbuk-do, Republic of Korea), which was collected from households, kitchens, and local dining establishments. Tofu by-products were gathered at a local tofu factory, formed as soybean curd residue (Kang-reung, Kangwon-do, Republic of Korea). Vegetables were gathered at a local agricultural market (Chuncheon, Kangwon-do, Republic of Korea), containing cabbage, lettuce, carrot, onion, and garlic. In total, 20 kg (DM-based) of substrate was added to each box. The nutrient composition of each substrate is shown in Table 1. To prevent drowning, larvae were reserved on 600 g of sawdust on the substrates.

### 2.4. Chemical Composition Analysis

The dry matter contents (DM), crude protein (CP), ether extract (EE), crude ash (Ash), and chitin contents of the harvested larvae from each treatment were evaluated using the Association of Official Analytical Chemists’ [22] methods. Fresh larvae were weighed and then dried in an oven at 85 °C for three days and reweighed to determine the DM content (using method 930.15 of the AOAC [22]). AOAC official method 990.03 was used to determine the protein content in samples using the Kjeldahl method. This method involves digesting the sample with sulfuric acid to release nitrogen and then converting the nitrogen to ammonia. The ammonia is then distilled and titrated to determine the amount of nitrogen present in the sample, which is then used to calculate the protein content [22]. AOAC official method 990.15 was used to determine the ether extract content in the samples. This method involves extracting the lipid content of the sample with a solvent and then evaporating the solvent to obtain the lipid content [22]. The gross energy of the BSF larvae was determined using a bomb calorimeter (Model 1261, Parr Instrument Co., Moline, IL, USA). The remaining samples were stored at −20 °C for future analysis.

### 2.5. Larval Convergence Efficiency Evaluation

To evaluate larval convergence efficiency from each botanical waste, the bioconversion rate (BCR), waste reduction rate (WR), protein conversion rate (PCR), lipid conversion rate (LCR), protein yield (PY), and lipid yield (LY), as well as the mass distribution pattern, were evaluated by applying the following equations:

BCR % = (DM_F_ − DM_I_)/DM_S_ × 100

where DM_F_ represents the DM of the BSFs at d 14, DM_I_ represents the DM of the BSFs at initiation, and DM_S_ represents the total DM of the substrate.

WR % = (DM_S_ − DM_R_)/DM_S_ × 100

where DM_S_ represents the total DM of the substrate; DM_R_ represents residual substrate DM.

PCR % = (DM_F_ × Protein_L_ %)/DM_S_ × 100

where DM_F_ represents the DM of the BSFs at d 14; DM_S_ represents the total DM of the substrate.

LCR % = (DM_F_ ×EE_L_ %)/DM_S_ × 100

where DM_F_ represents the DM of the BSFs at d 14; DM_S_ represents the total DM of the substrate.

PY kg/c = g of DM_F yield_ × CP_L_ %

where DM_F yield_ represents the DM of BSFs at harvest.

LY kg/c = g of DM_F yield_ × EE_L_ %

where DM_F yield_ represents the DM of the BSFs at harvest; c represents the clutch (50,000).

### 2.6. Fatty Acid Composition 

The fatty acid content was determined using a method described by Georgescu et al. [21]. To analyze the content, 10 g of larvae/diet were collected, kept for 24 h on a clean substrate to empty the digestive tract, washed with 70% ethanol to remove impurities, and stored at −80 °C. The extraction and identification of fatty acid methyl esters (FAMEs) from BSF larva fats were performed through gas chromatography with mass spectrometry detection, following the standards set by AOAC-969.33 [22], ISO 3657: 2002, ISO 12966-2: 2011, and ISO 12966-2: 2017. The process involved the saponification of the fat (using methanolic sodium hydroxide solution 0.5 M at 210 °C in a sand bath with a reflux rate of 1 drop/s), followed by esterification with a 15% vol boron trifluoride catalyst and cooling with hexane. The analysis was carried out on a Perkin Elmer chromatographic system with a mass spectrometer detector (GC-MS) consisting of a Clarus 680 gas chromatograph and a Clarus SQ8T quadrupole mass spectrometer. The system used an Elite-Wax chromatographic column with a stationary polar phase polyethylene glycol (PEG) of 30 m in length, an internal diameter of 0.25 mm, and a film thickness of 1.0 µm. The parameters used included an injection port temperature of 220 °C, an injected sample volume of 1.0 µL, a helium carrier gas flow rate of 1.5 mL/min, and a splitting ratio of 40:1. The MS had a transfer line temperature of 150 °C, a source temperature of 150 °C, a multiplier of 1500, and a solvent delay of 0–1.5 min. The determination of fatty acid concentration was performed by comparing the relative retention time of FAMEs to the certified standard Mix FAME Supelco 37. The individual fatty acid concentration was expressed as a percentage of the total identified FAMEs. The ratios between total saturated fatty acids (Σ SFA), total monounsaturated (Σ MUFA), total polyunsaturated (Σ PUFA), and total unsaturated fatty acids (Σ UFA) were calculated as follows: Σ PUFA/Σ SFA, Σ MUFA/Σ SFA, Σ UFA/Σ SFA, n-6 (fatty acids from the n-6 series), and n-3 (fatty acids from the n-3 series).

### 2.7. Statistical Analysis

The data were analyzed using the General Linear Model procedure of SAS (version 9.4, 1996, SAS Inst. Inc., Cary, NC, USA). The differences in growth performance, conversion efficiency, nutrient composition, and fatty acid concentrations between the treatments were analyzed using a one-way ANOVA test. The Tukey test was used to compare the means of the treatments, and a *p*-value less than 0.05 was considered statistically significant. A *p*-value less than 0.1 was considered a tendency. The results are presented as the mean ± standard deviation.

## 3. Results

### 3.1. Larvae Growing Performance

The effects of different botanical waste substrates on the larval growth performance of BSFs are shown in Table 2. The tofu by-product treatment showed the greatest weight at d 14 and the harvest period compared with the food waste and vegetable treatments (*p* < 0.05). Moreover, the BSF larval weight was greater in the food waste treatment compared with the vegetable treatment at d 14 and harvest. The larval length increased in the food waste and tofu by-product treatments (*p* < 0.05); however, larval width was not affected by the treatments. The vegetable treatment showed the longest development period (*p* < 0.05). The larval yield result was greater in the vegetable treatment (*p* < 0.05) compared with the food waste.

### 3.2. Nutrient Composition of BSF Larvae

The effects of different botanical waste substrates on the nutrient composition of BSFs are shown in Table 3. The tofu by-product treatment showed a greater percentage of DM compared with the food waste and vegetable treatments. The CP content increased in BSFs fed with tofu by-products, and the EE content was greater in the food waste and tofu by-product treatments compared with the vegetable treatment. No change was observed in the GE, ash, and chitin contents.

### 3.3. Larval Convergence Efficiency

The effects of different botanical waste substrates on the larval convergence efficiency of BSFs are shown in Table 4. The BCR was higher in the tofu by-product treatment (*p* < 0.05) compared with the vegetable and food waste treatments. A higher BCR was observed in BSFs fed food waste compared with vegetables. The WR was the highest in the tofu by-product treatment (*p* < 0.05). Moreover, the WR was higher in the food waste treatment (*p* < 0.05) compared with the vegetable treatment. The PCR was the highest in the vegetable treatment (*p* < 0.05) and was higher in the food waste treatment compared with the tofu by-product treatment. The LCR was the highest in the vegetable treatment (*p* < 0.05) and was higher in the tofu by-product treatment compared with the food waste treatment. The PY and LY were greatest in the tofu by-product treatment (*p* < 0.05) and increased in the food waste treatment compared with the vegetable treatment.

### 3.4. Fatty Acid Composition

The effects of different botanical waste substrates on the fatty acid composition of BSFs are shown in Table 5. The C12:0 (lauric acid) content increased in BSFs fed tofu by-products compared with the food waste treatment. The concentration of C16:1 was the highest in the tofu by-product treatment. The C18:1 (oleic acid) and C18:3n3 (α-linolenic acid) contents were higher in BSFs fed tofu by-products compared with the vegetable treatment. There was no difference between the treatments for the content of other fatty acids.

## 4. Discussion

The benefits of using BSFs for organic WR are due to their high conversion ability, converting organic waste into their body mass [7,13,15,23]. The growth of BSFs can be enhanced by the contents of organic waste [4,7,19]. In this study, food waste and tofu by-products showed higher growth performance, as both contain balanced nutrients. Our results are in agreement with previous studies [17,24], in that larvae reared on well-balanced protein and carbohydrates improved their growth and survivability. However, the tofu by-products showed the lowest survivability for the BSFs, which may be because of the high protein content, which may increase ammonia gas emissions. During the trial, tofu by-products were stored in a thick layer at room temperature, which potentially could cause the release of noxious gasses. The current study also revealed that the development time of BSF larvae differed based on the substrates. The larvae reared on vegetable substrates took over 20 days to develop. The vegetables included cabbage, lettuce, radish, and carrot, with low levels of protein and fat but high levels of fiber. In general, high-fiber substrates can reduce the growth rate of BSF larvae, as they are less digestible compared with low-fiber substrates [25]. The results of this study are in line with previous reports showing a longer growth period for BSF larvae in vegetable-containing substrates [13,19,21].

Black soldier fly larvae are emerging as a promising protein source for animal feed because of their high feed-to-protein conversion rate, which has the potential to reduce the cost of animal feed [4,19]. Using food waste and vegetables as a feed source for BSF larvae is an attractive option for promoting sustainable agriculture [6,13,19]. The nutritional composition of BSF larvae is affected by the composition of the rearing substrate, which may vary in terms of macronutrient and mineral content [3]. Several studies have reported that BSF larvae are rich in CP and EE [1,9,26,27]. However, the composition of the rearing substrate can also impact nutrient accumulation in the larvae. In the present study, we investigated the effect of different substrates, including food waste, tofu by-products, and vegetables, on the nutritional composition of BSF larvae. Our results showed that the tofu by-product treatment resulted in larvae with higher fat content compared with the food waste treatment. This may be attributed to the faster development of BSF larvae reared on tofu by-products, which resulted in a higher body composition until the early prepupae stage. On the other hand, the vegetable treatment had lower DM, CP, and EE contents, likely because of its high moisture content but low CP and EE [13,21]. The ash content of BSFs can vary depending on factors such as season, location, and ingredients [14,19]. Some studies have reported low ash content in BSFs reared on vegetable sources [19]. However, in this study, there was no difference in ash content across the substrates, with the vegetables having the lowest ash content compared with food waste and tofu by-products. Chitin, a polysaccharide composed of a long-chain polymer of N-acetylglucosamine, is a major structural component of insect cuticles, has beneficial properties, and is an immune-amplifying and antimicrobial supplement [5], but it can also negatively affect digestibility in monogastric animals. In the present study, we found no difference in chitin levels across the substrates. Chitin levels are influenced by factors such as species, substrate, and stage [3,5]. In this study, there was no difference in the chitin level across the substrates. We also evaluated the BCR, PCR, and LCR of the BSF larvae. Our results showed that the tofu by-product treatment had the highest BCR, PCR, and LCR. These findings suggest that tofu by-products, as a substrate for BSF larvae, have the potential to be a sustainable and cost-effective protein source for animal feed. A similar result was reported by Rehman et al. [15], where an increase in the tofu by-product ratio led to a higher BCR.

In our study, the BSF larvae reared on vegetable substrate had the lowest BCR. This is consistent with the findings of Lalander et al. [13], who reported that BSF larvae reared on vegetable-based substrates showed lower BCR compared with those reared on organic waste substrates. Using vegetable-based substrates can result in lower protein content in the larvae because of their low protein content compared with food waste [25]. This can lead to lower protein conversion rates, which is the amount of protein produced per unit of substrate consumed by the larvae [28]. Studies have also shown that vegetable-based substrates in BSF diets can have a negative impact on WR and BCR [13,25]. However, vegetable and fruit sources can increase the BCR of BSFs if the substrate contains a high amount of protein (19.66%) [28]. WR is the amount of waste reduction per unit of biomass produced by larvae [15,16]. In terms of WR, vegetable-based substrates have been shown to be less efficient compared with food waste [25]. A higher WR indicates that the larvae are more efficient in converting feed into biomass [14]. In a study by Singh et al. [25], feeding BSF larvae a diet of vegetable waste resulted in a lower WR compared with restaurant waste, fruit waste, and mixed food waste, indicating that vegetable-based substrates require more feed to produce the same amount of biomass regarding the relatively high fiber content. The WR was highest for the tofu by-product treatment, followed by the food waste treatment. This could help explain the difference in maturation days between the BSF larvae. The vegetable treatment had the highest PCR; however, the highest PY was observed in the tofu by-product treatment. This can be attributed to the 12-day harvest period, during which the tofu by-product treatment showed the highest body mass. PCR is the amount of protein consumed per unit of biomass produced, while PY is the total protein biomass produced by the larvae [11,29]. The lower PCR in the tofu by-product treatment can also be attributed to the relatively low availability of carbohydrates and lipids in the substrate, leading the larvae to consume more protein in order to meet their energy requirements. This is supported by the findings of a study by Seyedalmoosavi et al. [12], who reported that protein content in the diet is a major determinant of the PCR in BSF larvae. Furthermore, the protein content and composition of the substrate can also impact the quality of the protein produced by the larvae. Hopkins et al. [16] found that feeding BSF larvae a diet of vegetable waste resulted in a higher PCR compared with a diet of fish waste. This suggests that vegetable-based substrates may lead to higher protein efficiency in the larvae.

The vegetable treatment also resulted in a higher LCR compared with the food waste and tofu by-product treatments; however, the tofu by-product treatment had a higher LY compared with the food waste and vegetable treatments. The higher LCR in the vegetable-based treatment can be attributed to the relatively low availability of lipids in the substrate [12], leading the larvae to retain dietary lipids more efficiently. In contrast, the higher LY in the tofu by-product treatment may be because of the relatively high lipid content in the substrate. In summary, our study demonstrates that the nutritional composition of BSF larvae can be influenced by the composition of the rearing substrate. The use of tofu by-products as a substrate for BSF larvae can result in larvae with higher fat content and a more favorable BCR. On the other hand, the vegetable treatment showed a greater PCR and LCR efficiency. These findings highlight the importance of using the substrates in combination to increase the conversion ratio efficiency of BSF larvae as a sustainable protein source for animal feed.

In our study, we found that the content of α-linolenic acid was greater in BSFs fed tofu by-products compared with those fed with vegetables. The fatty acid composition of BSF larvae can vary depending on various factors, such as the composition of their rearing substrate, the stage of their development, and the environmental conditions [1,30,31]. Previous studies have shown that BSF larvae are a rich source of unsaturated fatty acids, such as linoleic acid and oleic acid, which are considered beneficial for animal health [21]. Additionally, some studies have also reported the presence of essential fatty acids, such as alpha-linolenic acid, in BSF larvae.

Our results are in agreement with previous studies that confirmed the high content of oleic acid and lauric acid in BSFs [1,30,32]. The relatively high content of lauric acid, oleic acid, and linoleic acid in our study suggests that tofu by-products and vegetable waste are both good substrates for producing BSF larvae with a favorable fatty acid profile. Other studies have reported that BSF meal fed with animal waste or lipid-rich diets has higher lauric acid content compared to BSF meal reared on plant-based diets [30]. Conversely, plant-based diets can result in higher oleic acid and linoleic acid content in BSF meal [31]. Therefore, the fatty acid profile of BSF larvae can be influenced by the type of food waste or organic matter they are reared on, as well as the stage of their development. Further research is needed to better understand the impact of these factors on the fatty acid profile of BSF larvae and how it can be optimized for various applications, such as animal feed or human consumption. Additionally, the optimal dietary requirements for producing BSF larvae with a favorable fatty acid profile for human consumption are not yet fully understood. As such, future research should focus on determining the optimal substrate and feeding regimen to produce BSF larvae with a desirable fatty acid profile that meets the nutritional requirements of humans. Overall, our findings contribute to the growing body of research on the potential use of BSF larvae as a sustainable and nutrient-rich protein source for animal feed and human consumption.

## 5. Conclusions

Supplying different substrates shows the potential for growth performance and nutrient and fatty acid composition enhancement compared with general methods and food waste substrates. In particular, tofu by-products show the greatest values in growth performance, protein and lipid contents, convergence efficiency, and some essential fatty acids for livestock animals. However, vegetables show a lower performance compared with food waste, except for fatty acid composition results. Thus, tofu by-products are the best option for BSF rearing and product quality, and vegetables can be a cheap source for enhancing essential fatty acid composition in livestock animals.

## Figures and Tables

**Table 1:** Nutrient composition of botanical waste used in the trial; dry matter-based.

Substrates	Food Waste	Tofu By-Product	Vegetables
Dry matter, %	17.72	24.1	11.1
Crude protein, %	20.2	29.2	11.2
Ether extract, %	19.36	13.1	8.6
Ash, %	7.66	7.42	5.9

**Table 2:** Effects of different botanical waste on the larval growth performance of BSF ^1^ larvae.

Specification		Food Waste	Tofu By-Product	Vegetables	*p*-Value
Larval weight	d 12.5	165.8 ± 1.42 ^b^	199.9 ± 1.58 ^a^	105.4 ± 0.65 ^c^	0.009
	At harvest	192.9 ± 1.66 ^b^	199.9 ± 1.58 ^a^	179.1 ± 1.12 ^c^	<0.001
Larval length, mm		15.88 ± 0.18 ^a^	16.25 ± 0.27 ^a^	14.61 ± 0.22 ^b^	0.002
Larval width, mm		2.93 ± 0.03	3.00 ± 0.02	2.93 ± 0.02	0.081
Development, d		14.0 ± 0.74 ^b^	12.5 ± 0.66 ^b^	20.4 ± 1.08 ^a^	<0.001
Larvae yield, %		79.7 ± 0.87 ^ab^	77.5 ± 0.68 ^b^	82.2 ± 0.71 ^a^	0.001

^1^ BSF, black soldier fly. ^a,b,c^ means that different superscripts in the same row differ significantly (*p* < 0.05).

**Table 3:** Chemical composition of BSF ^1^ reared on 3 botanical waste substrates based on dry matter (DM).

Specification	Food Waste	Tofu By-Product	Vegetables	*p*-Value
DM, %	40.99 ± 1.56 ^ab^	42.94 ± 1.48 ^a^	37.98 ± 1.11 ^b^	0.019
Gross energy, MJ	18.25 ± 0.21	18.43 ± 0.30	17.69 ± 0.21	0.053
Crude protein, %	39.52 ± 0.94 ^b^	43.54 ± 0.88 ^a^	37.65 ± 0.96 ^b^	<0.001
Ether extract, %	38.07 ± 0.78 ^a^	37.03 ± 0.81 ^a^	34.45 ± 0.48 ^b^	0.001
Ash, %	7.16 ± 0.39	7.28 ± 0.43	7.77 ± 0.41	0.421
Chitin, %	8.06 ± 0.54	8.68 ± 0.62	7.12 ± 0.52	0.080

^1^ BSF, black soldier fly. ^a,b^ means that different superscripts in the same row differ significantly (*p* < 0.05).

**Table 4:** Convergence efficiency of BSF ^1^ larvae reared on 3 botanical waste substrates.

Specification ^2^	Food Waste	Tofu By-Product	Vegetables	*p*-Value
BCR, %	12.10 ± 0.34 ^b^	13.58 ± 0.29 ^a^	9.88 ± 0.21 ^c^	<0.001
WR, %	66.54 ± 0.77 ^b^	71.35 ± 0.46 ^a^	51.90 ± 0.76 ^c^	<0.001
PCR, %	11.30 ± 0.28 ^b^	8.69 ± 0.19 ^c^	21.44 ± 0.46 ^a^	<0.001
LCR, %	11.35 ± 0.28 ^c^	16.48 ± 0.35 ^b^	25.55 ± 0.55 ^a^	<0.001
Protein yield, kg/c	1.25 ± 0.03 ^b^	1.45 ± 0.03 ^a^	1.05 ± 0.02 c	<0.001
Lipid yield, kg/c	1.20 ± 0.03 ^b^	1.23 ± 0.03 ^a^	0.96 ± 0.02 ^c^	<0.001

^1^ BSF, black soldier fly. ^2^ BCR, bioconversion rate; WR, waste reduction rate; PCR, protein conversion rate; LCR, lipid conversion rate; PY, protein yield; LY, lipid yield; kg/c, kg per clutch (50,000). ^a,b,c^ means that different superscripts in the same row differ significantly (*p* < 0.05).

**Table 5:** Fatty acid composition of BSF ^1^ reared on 3 botanical waste substrates.

Specification ^2^	Food Waste	Tofu By-Product	Vegetables	SEM ^3^	*p*-Value
Fatty acid, % of FAME					
C10:0	1.584 ± 0.099	1.576 ± 0.203	1.581 ± 0.134	0.068	0.392
C12:0	20.972 ± 0.910 ^b^	22.300 ± 1.276 ^a^	21.431 ± 0.839 ^ab^	0.420	0.013
C14:0	2.371 ± 0.173	2.352 ± 0.367	2.36 ± 0.219	0.115	0.386
C14:1	0.168 ± 0.015	0.168 ± 0.034	0.168 ± 0.021	0.011	0.996
C15:0	0.058 ± 0.005	0.063 ± 0.010	0.058 ± 0.007	0.003	0.764
C15:1	0.066 ± 0.004	0.066 ± 0.010	0.068 ± 0.007	0.003	0.758
C16:0	8.858 ± 0.175	8.873 ± 0.388	8.860 ± 0.233	0.125	0.539
C16:1	3.222 ± 0.281 ^b^	3.378 ± 0.615 ^a^	3.250 ± 0.406 ^b^	0.035	0.855
C18:0	1.843 ± 0.080	1.832 ± 0.168	1.835 ± 0.116	0.057	0.478
C18:1	25.966 ± 0.427 ^ab^	26.558 ± 1.368 ^a^	24.138 ± 1.334 ^b^	0.483	0.031
C18:2n6	25.003 ± 0.786	25.467 ± 0.656	25.319 ± 0.487	0.280	0.251
C18:3n3	2.380 ± 0.049 ^ab^	2.431 ± 0.100 ^a^	2.301 ± 0.113 ^b^	0.037	0.027
C18:4	0.508 ± 0.037	0.502 ± 0.078	0.506 ± 0.054	0.026	0.967
C20:1	0.408 ± 0.018	0.415 ± 0.036	0.412 ± 0.025	0.012	0.815
C20:4n6	0.322 ± 0.011	0.321 ± 0.022	0.322 ± 0.014	0.007	0.991
C20:5n3	1.723 ± 0.026	1.722 ± 0.053	1.722 ± 0.035	0.018	0.998
C22:6n3	0.225 ± 0.010	0.221 ± 0.020	0.219 ± 0.014	0.007	0.896

^1^ BSF, black soldier fly. ^2^ FAME, fatty acid methyl ester. ^a,b^ means that different superscripts in the same row differ significantly (*p* < 0.05). ^3^ standard error of the mean.

## Data Availability

Upon reasonable request, the datasets of this study can be made available from the corresponding author.
